# Transcriptome Reveals Roles of Lignin-Modifying Enzymes and Abscisic Acid in the Symbiosis of *Mycena* and *Gastrodia elata*

**DOI:** 10.3390/ijms22126557

**Published:** 2021-06-18

**Authors:** Li-Ying Ren, Heng Zhao, Xiao-Ling Liu, Tong-Kai Zong, Min Qiao, Shu-Yan Liu, Xiao-Yong Liu

**Affiliations:** 1 College of Plant Protection, Jilin Agricultural University, Changchun 130118, China; renly@im.ac.cn; 2 Engineering Research Center of Edible and Medicinal Fungi, Ministry of Education, Jilin Agricultural University, Changchun 130118, China; 3 State Key Laboratory of Mycology, Institute of Microbiology, Chinese Academy of Sciences, Beijing 100101, China; zhaoheng181@mails.ucas.ac.cn (H.Z.); liuxl19@im.ac.cn (X.-L.L.); 4 College of Life Sciences, University of Chinese Academy of Sciences, Beijing 100049, China; 5 Key Laboratory for Forest Resources Conservation and Utilization in the Southwest Mountains of China, Ministry of Education, Southwest Forestry University, Kunming 650224, China; zongtongkai@im.ac.cn; 6 State Key Laboratory for Conservation and Utilization of Bio-Resources in Yunnan, Yunnan University, Kunming 650091, China

**Keywords:** *Gastrodia elata*, *Mycena* sp., POD, laccase, ABA, orchid germination

## Abstract

*Gastrodia elata* is a well-known medicinal and heterotrophic orchid. Its germination, limited by the impermeability of seed coat lignin and inhibition by abscisic acid (ABA), is triggered by symbiosis with fungi such as *Mycena* spp. However, the molecular mechanisms of lignin degradation by *Mycena* and ABA biosynthesis and signaling in *G. elata* remain unclear. In order to gain insights into these two processes, this study analyzed the transcriptomes of these organisms during their dynamic symbiosis. Among the 25 lignin-modifying enzyme genes in *Mycena*, two ligninolytic class II peroxidases and two laccases were significantly upregulated, most likely enabling *Mycena* hyphae to break through the lignin seed coats of *G. elata*. Genes related to reduced virulence and loss of pathogenicity in *Mycena* accounted for more than half of annotated genes, presumably contributing to symbiosis. After coculture, upregulated genes outnumbered downregulated genes in *G. elata* seeds, suggesting slightly increased biological activity, while *Mycena* hyphae had fewer upregulated than downregulated genes, indicating decreased biological activity. ABA biosynthesis in *G. elata* was reduced by the downregulated expression of 9-cis-epoxycarotenoid dioxygenase (NCED-2), and ABA signaling was blocked by the downregulated expression of a receptor protein (PYL12-like). This is the first report to describe the role of NCED-2 and PYL12-like in breaking *G. elata* seed dormancy by reducing the synthesis and blocking the signaling of the germination inhibitor ABA. This study provides a theoretical basis for screening germination fungi to identify effective symbionts and for reducing ABA inhibition of *G. elata* seed germination.

## 1. Introduction

Orchids are perennial monocotyledons, the third largest family in angiosperms. These plants are mainly terrestrial, epiphytic and saprophytic, and have ornamental, medicinal, and economic value [1]. *Gastrodia elata* Bl. is a widespread orchid that contains gastrodin, vanillanol, and other medicinal ingredients that play important roles in lowering blood pressure, increasing blood flow of the heart and brain, decreasing inflammation, and improving body immunity [2,3]. Lacking roots and leaves, *Gastrodia elata* is heterotrophic and depends on the symbiotic fungi *Mycena* spp. and *Armillaria mellea* (Vahl) P. Kumm to supply necessary nutrients for seed germination, tuber formation, flowering, and fruiting [4,5,6,7]. *Mycena* spp. promote seed germination and protocorm formation [7], and *Armillaria mellea* supports the other stages of its lifecycle [8].

Two potential reasons have been proposed to explain the poor germination of terrestrial orchid (including *Gastrodia elata*) seeds [9,10,11]: the restriction of water uptake due to the impermeable seed coat [12,13] and the presence of germination inhibitors, such as phenolics and abscisic acid (ABA) [14,15]. The seed coats of *Cypripedium calceolus* and *Epipactis palustris* are composed of lignin, which forms a hydrophobic barrier and prevents seeds from germinating. Even when they were cultivated together with fungi, seed germination was not significantly improved until their coat was perforated by Ca(ClO)_2_ [16,17]. Thus, degrading the lignin coat is key to improving the seed germination rate. Fungal lignin-modifying enzymes responsible for the degradation function are divided into three categories: laccases, dye-peroxidases (DyP), and ligninolytic class II peroxidases (POD, including lignin peroxidase, manganese-dependent peroxidase, and versatile peroxidase) [18,19]. The seed coat of *G. elata* is only composed of lignin [20], and the lignin-degrading ability of the germination-promoting fungus *Mycena* is presumably the key to their symbiosis. However, to date, studies on the molecular mechanism of lignin degradation in *Mycena* have not been conducted.

The plant hormone abscisic acid (ABA) is a seed germination inhibitor [15]. An increase in ABA content inhibited seed germination in *Dactulorhiza maculate* [14], while a decrease promoted seed germination in *Cypripedium formosanum* [21]. Two pathways have been suggested for ABA biosynthesis. One involves its direct formation from a C15 precursor, farnesyl pyrophosphate, and this type of pathway occurs mainly in fungi. The other is known as the carotenoid pathway, which is predominant in higher plants. A C40 xanthophyll carotenoid is used as a precursor to form ABA through a series of redox reactions by zeaxanthin epoxidase (ZEP), 9-cis-epoxycarotenoid dioxygenase (NCED), short-chain dehydrogenase/reductase (ABA2), and abscisic aldehyde oxidase (AAO3) [22,23,24]. In addition, the ABA signaling pathway also regulates seed germination [25]. In the core steps, ABA binds to PYR/PYL/RCAR receptors and triggers a conformational change. The receptor–ABA complex further binds to a type 2C protein phosphatase (PP2C), activating kinases such as the sucrose nonfermenting1-related protein kinase 2 (SnRK2). In turn, these kinases activate an ABA-responsive element-binding factor (ABF), which binds to target promoters and induces the expression of ABA response genes [26,27,28,29,30,31]. However, the molecular mechanisms of ABA synthesis and signal pathway inhibition during the germination of *Gastrodia elata* seeds remain unclear.

In this study, genome and transcriptome data were used to analyze the lignin-modifying enzyme genes of the *Mycena* sp. strain WM to understand its lignin degradation ability. The transcriptome was used to analyze the dynamic process of symbiosis between *Gastrodia elata* and *Mycena* and thereby clarify the molecular mechanisms of ABA biosynthesis and its signal transduction pathway.

## 2. Results

### 2.1. Development of Symbiotic Protocorms

**Figure 1:**
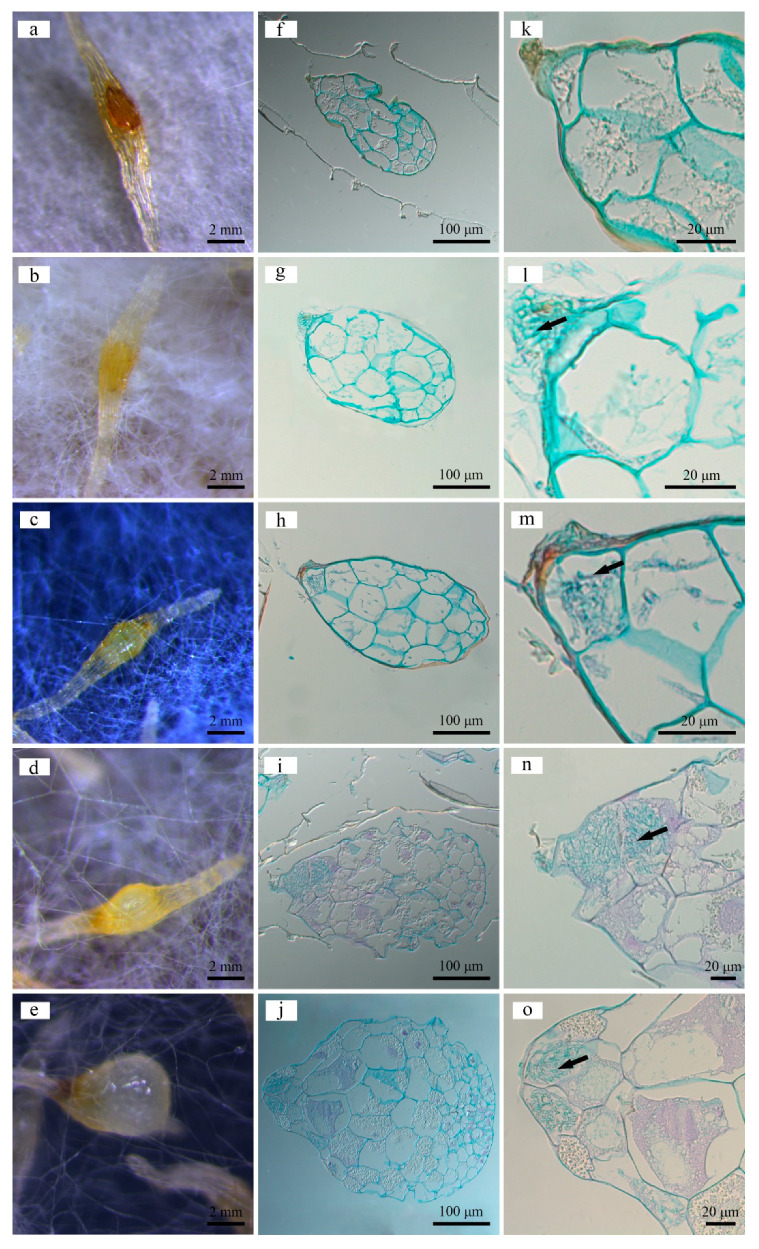
Macroscopic and histological observations of coculture of *Gastrodia* *elata* seeds and *Mycena* sp. WM captured on the initial (**a**,**f**,**k**), 5th (**b**,**g**,**l**), 10th (**c**,**h**,**m**), 15th (**d**,**i**,**n**), and 20th (**e**,**j**,**o**) days. (**a**–**e**) Macroscopic view under a stereoscope; (**f**–**j**) histological sections of *G. elata* seed under a microscope; (**k**–**o**) partial enlarged details of the picture in b showing base cells. Arrows indicate the *Mycena* hyphae invading the seed cell.

Intracellular fungal hyphae in symbiotically germinating seeds were stained with the glycogen PAS kit (Figure 1). Initially, the *Gastrodia elata* seeds were brown. On the fifth day of coculture with *Mycena* sp. WM, they became yellow. Fungal hyphae broke through the seed coat and were concentrated in the residue of suspensor cells. On the 10th day, the protocorms expanded, and fungal hyphae invaded the suspensor cells. On the 15th day, protocorms burst through the seed coat, and the apical meristem divided and differentiated, while fungal hyphae invaded the outermost 1–2 layers of proembryo cells. On the 20th day, the protocorms further expanded and became dewdrop-shaped, and fungal hyphae remained in the outermost 1–2 layers of proembryo cells.

### 2.2. Genome Sequence Assembly and Annotation

A total of 23,038 Mb of clean data were obtained from next-generation sequencing in *Mycena* sp. WM. After statistical analysis and error correction, clean reads were assembled into 63,394 scaffolds to a final assembly of 166.99 Mb with an estimated coverage of 137 and a scaffold N50 of 4415 bp. A total of 40,275 gene models were predicted with 50.91% GC content. The predicted gene models were compared with six databases, resulting in a total of 19,648 coding genes, of which 1121 genes were annotated by COG, 1830 were annotated by SWISS-PROT, 15391 were annotated by NR, 8869 were annotated by GO (Figure S1a), 3483 were annotated by KEGG (Figure S1b), 445 were annotated by CAZy (Table S1), and 5024 were annotated by PHI (Table S2). PHI database annotation indicated that 56.93% of genes were classified as reduced virulence and loss of pathogenicity, while only 3.60% were related to increased pathogenicity (Table S2). The raw data are available in the BioProject repository of NCBI under the accession number PRJNA730200. The genome assembly of *Mycena* sp. WM was deposited in GenBank under the accession number JAHHPV000000000.

### 2.3. Transcriptome Sequence Assembly and Annotation

**Figure 2:**
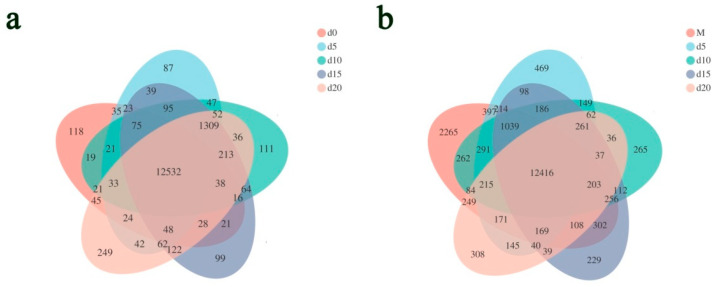
Venn diagrams of the expressed gene numbers on the initial (d0 or M), 5th (d5), 10th (d10), 15th (d15), and 20th (d20) days of the coculture. (**a**) *Gastrodia elata*; (**b**) *Mycena* sp. WM.

A total of 18 transcriptomes were obtained, including 3 of *Gastrodia elata* seeds, 3 of pure *Mycena* sp. WM culture, and 12 of their coculture. The clean data of each transcriptome related to *G. elata* were more than 6.42 Gb, with 92.69% of Q30. The mapping rates ranged from 18.93% to 96.54% (Table S3). In total, 19,845 expressed genes were detected across all 15 transcriptomes related to *G. elata*. The raw reads are available in the BioProject repository of NCBI under the accession number PRJNA729600. Approximately 86.04% of the expressed genes were annotated against the NR, SWISS-PROT, Pfam, COG, GO, and KEGG databases (Table S4). Venn analysis revealed that 12,532 genes were shared by the five germination time points (Figure 2a).

The clean data of each transcriptome related to *Mycena* sp. WM were more than 1.45 Gb, with 92.10% of Q30. The mapping rates ranged from 9.41% to 82.39% (Table S3). In total, 28,173 expressed genes were detected across all 15 transcriptomes related to *Mycena* sp. WM. The raw reads are available in the BioProject repository of NCBI under the accession number PRJNA729600. Approximately 20,486 (72.72%) of the expressed genes were annotated against the NR, SWISS-PROT, Pfam, COG, GO, and KEGG databases (Table S4). Venn analysis revealed that 12,416 genes were shared by the five germination time points (Figure 2b).

### 2.4. Differentially Expressed Genes

**Figure 3:**
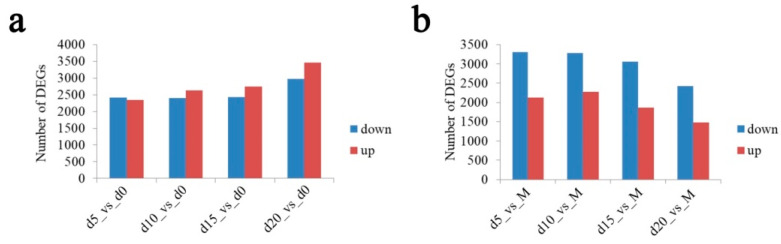
Bar charts of the number of up- and downregulated differentially expressed genes (DEGs) on the 5th (d5), 10th (d10), 15th (d15), and 20th (d20) days of the coculture compared with the initial values (d0 or M). (**a**) *Gastrodia elata*; (**b**) *Mycena* sp. WM.

Compared to the expression data of *Gastrodia elata* seeds, differentially expressed gene (DEG) analysis revealed fewer upregulated than downregulated genes on the 5th day of coculture, whereas more genes were upregulated than downregulated on the 10th, 15th, and 20th days (Figure 3a). GO functional annotation showed that DEGs were mainly concentrated in metabolic and cellular biological processes, membrane and cell parts in cellular components, and catalytic activity and binding in molecular functions (Figure S2). KEGG pathway enrichment analysis showed that DEGs were mainly classified as phenylpropanoid biosynthesis; plant hormone signal transduction; MAPK signaling pathway—plant; starch and sucrose metabolism; and glutathione metabolism (Figure S4).

Compared to the expression data from pure *Mycena* sp. WM culture, DEG analysis revealed that downregulated genes outnumbered upregulated genes on the 5th, 10th, 15th, and 20th days of coculture (Figure 3b). GO functional annotation analysis showed that DEGs were mainly concentrated in metabolic and cellular biological processes, membrane and cell parts in cellular components, and catalytic activity and binding in molecular function (Figure S3). KEGG pathway enrichment analysis showed that DEGs were mainly classified as ribosome biogenesis in eukaryotes; tryptophan metabolism; amino sugar and nucleotide sugar metabolism; spliceosome, arginine, and proline metabolism; ribosome; and glycine, serine, and threonine metabolism (Figure S5).

### 2.5. Fungal Lignin-Modifying Enzymes

The genomes of the four orchid mycorrhizal fungi, including *Mycena* sp. WM, were annotated by the CAZy database. There were some differences in the number of laccase and DyP genes, but the number of POD genes was significantly different (Table S5). As many as 12 POD genes were identified in the WM strain, but none were detected in *Sebacina vermifera*, *Tulasnella calospora* or *Ceratobasidium* sp. Histological observations showed that fungal hyphae broke through the seed coat on the fifth day of coculture. Therefore, a comparative transcriptome study of lignin-modifying enzyme genes was conducted between the hyphae of *Mycena* sp. WM in pure culture and those on the fifth day of coculture. Analysis of transcriptome expression of the 12 POD genes in the WM strain showed that 5 genes were upregulated (including 2 significantly upregulated, *p* < 0.05), 6 were downregulated, and 1 was unexpressed. The two significantly upregulated genes were GME42162_g and GME24208_g, with upregulation levels of 7.01- and 5.92-fold, respectively (Table 1). Analysis of transcriptome expression of the 11 laccase genes in the WM strain showed that 3 genes were upregulated, 7 were downregulated, and 1 was unexpressed. Two genes (GME40562_g and GME8664_g) were significantly upregulated (*p* < 0.05), with upregulation levels of 12.58- and 7.53-fold, respectively (Table 1). Other genes, including the two DyP genes, were not significantly up- or downregulated (Table 1).

### 2.6. ABA Biosynthesis and Signaling

**Figure 4:**
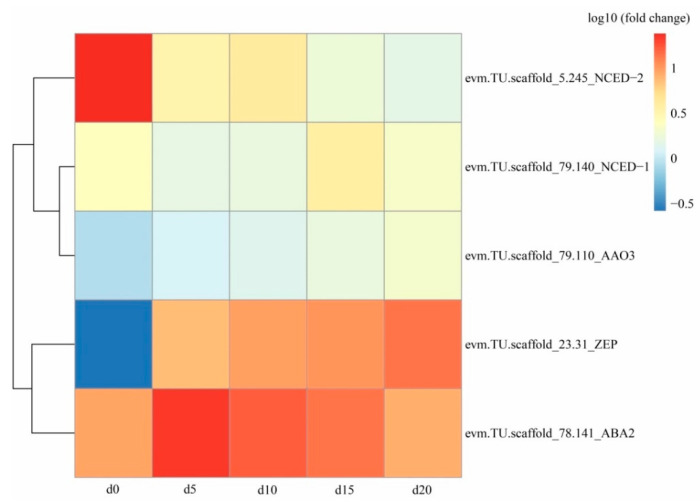
The heat map of expression levels of genes associated with abscisic acid (ABA) biosynthesis in *Gastrodia elata* on the initial (d0), 5th (d5), 10th (d10), 15th (d15), and 20th (d20) days of the coculture with *Mycena* sp. WM.

**Figure 5:**
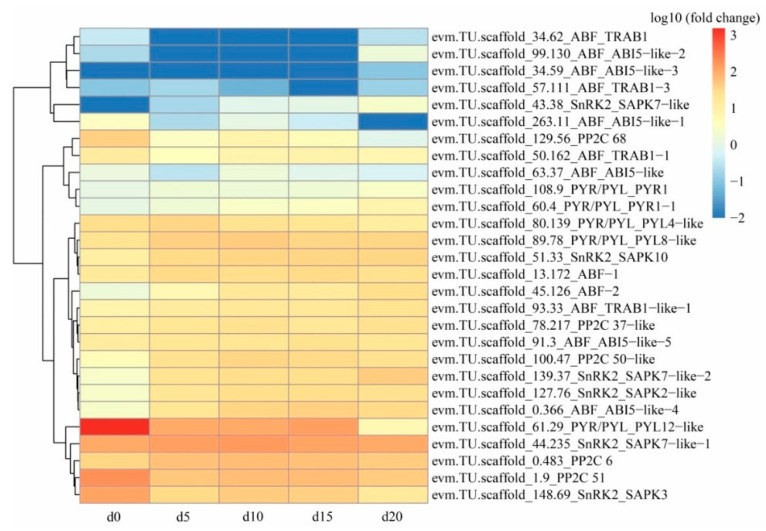
The heat map of expression levels of genes associated with abscisic acid (ABA) signal transduction in *Gastrodia elata* on the initial (d0), 5th (d5), 10th (d10), 15th (d15), and 20th (d20) days of the coculture with *Mycena* sp. WM.

The analysis of the dynamic expression levels of genes related to ABA biosynthesis showed that NCED-2 (evm.TU.scscaffold_5.245), the most critical enzyme, was significantly downregulated from the 5th to 20th day of coculture (Figure 4). In terms of ABA signal transduction, the ABA receptor PYL12 (evm.TU.scaffold_61.29) was markedly downregulated (Figure 5).

### 2.7. Gene Expression Detected via qRT-PCR

**Figure 6:**
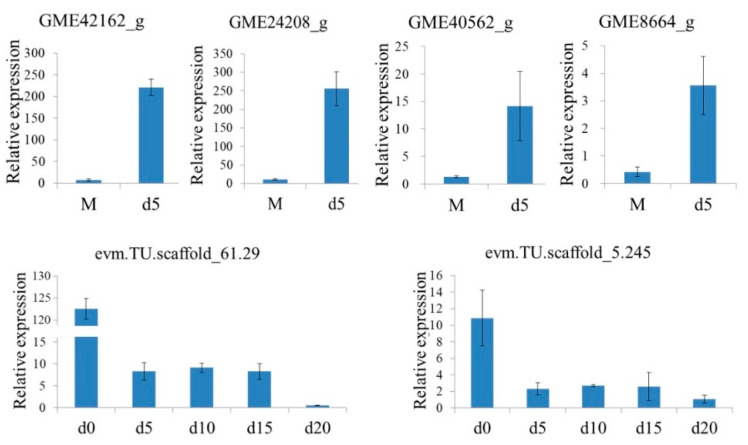
The RT-qPCR analysis of two upregulated peroxidase (POD) genes (GME42162_g and GME24208_g) and two upregulated laccase genes (GME40562_g and GME8664_g) in *Mycena* sp. WM and two downregulated abscisic acid (ABA)-related genes (NCED-2, evm.TU.scscaffold_5.245 and PYL12-like, evm.TU.scaffold_61.29) at different time points (beginning, d0; 5th day, d5; 10th day, d10; 15th day, d15; 20th day, d20) of their coculture. Bars represent the standard error of the mean, SE (n = 9).

To confirm the reliability of RNA-seq data, quantitative reverse transcription PCR (RT-qPCR) analysis was performed on the four upregulated lignin-modifying enzyme-related genes (GME42162_g, GME24208_g, GME40562_g, and GME8664_g), one gene involved in ABA biosynthesis (NCED-2, evm.TU.scscaffold_5.245), and one gene involved in ABA signaling (PYL12-like, evm.TU.scaffold_61.29). The results show (Figure 6) that the four lignin-modifying enzyme-related genes were weakly expressed in pure hyphae but highly expressed in coculture on the fifth day. The two genes involved in ABA biosynthesis and signal transduction were highly expressed in seeds and weakly expressed in symbiotic developmental protocorms. These data reveal similar trends to those obtained from the transcriptomic data.

## 3. Discussion

This study annotated genes in the orchid mycorrhizal fungi *Tulasnella calospora*, *Sebacina vermifera* and *Ceratobasidium* sp., and no POD genes were identified, confirming the finding by Miyauchi et al. [32]. However, as many as 12 POD genes were found in the genome of *Mycena* sp. WM. This implies that *Mycena* sp. WM might be an extraordinary symbiont. Transcriptome analysis was performed on the 12 POD genes on the fifth day of coculture compared to pure culture (d5 vs. M). The results reveal that five manganese-dependent peroxidases were upregulated, of which two were significantly upregulated (*p* < 0.05, Table 1), i.e., the expression levels of GME42162_g (short manganese peroxidase, MnP-short) and GME24208_g (short manganese peroxidase, MnP-short) increased by 7.01- and 5.92-fold, respectively. Manganese-dependent peroxidase (MnP) was reported to be key to ligninolysis in white-rot fungi [33,34]. In *Phlebia radiata*, MnP1-long, MnP2-long, and MnP3-short genes were highly upregulated, promoting colonization on spruce wood [35]. When *Pleurotus ostreatus* grew on a natural lignocellulosic solid substrate of cotton stalks, the MnP-short gene was predominately expressed. When deactivated, its ability to degrade lignin decreased [36]. Our results show that when *Mycena* broke through the seed coat, the upregulation of MnP-short genes was conducive to the invasion of *Gastrodia elata* seeds by hyphae and the establishment of a symbiotic relationship. Transcriptome analysis was performed on the 11 laccase genes on the fifth day of coculture compared to pure culture (d5 vs. M). The results reveal that three laccases were upregulated, of which two were significantly upregulated (*p* < 0.05, Table 1), i.e., the expression levels of GME40562_g (laccase-1) and GME8664_g (laccase) increased by 12.58- and 7.53-fold, respectively. Laccases produced in variable quantities by basidiomycete and saprophytic fungi are the most important components of the ligninolytic complex and have been commercially used for the delignification of woody materials [37,38,39,40]. After treating the seeds of *Anacamptis morio* (Orchidaceae) with laccase, Pierce et al. observed an increase in lignin degradation and water uptake, which in turn significantly promoted the germination of the seeds [41]. Our results show that the expression of laccase genes was significantly upregulated to produce more laccase for degrading the lignin seed coat. Taken together, these could lead to a conclusion that MnP and laccase are responsible for lignin degradation when *Mycena* breaks through the seed coats of *Gastrodia elata*. In order to validate the reliability of transcriptome data, the fold changes of RNA-seq data were compared with those of RT-qPCR data. The findings are consistent with transcriptome results; the two MnP genes and two laccase genes were weakly expressed in pure hyphae but highly expressed in hyphae when symbiosis with *G. elata* seeds was formed on the fifth day.

The genome size of *Mycena* sp. WM was 166.99 Mb with 50.91% GC content and 40,275 predicted gene models. Among them, 5024 genes were annotated in the PHI database, among which more than half were linked to reduced virulence (47.69%, Table S2) and loss of pathogenicity (9.24%, Table S2). This presumably contributes to the successful establishment of symbiosis between the fungus *Mycena* sp. WM and *Gastrodia elata*, as Jansen et al. [42] noted that virulence attenuation was related to the increased adaptability of the pathogen to the host. After their symbiotic relationship was established, the biological activity in *G. elata* protocorms was slightly increased compared to that of pure seeds, with more upregulated than downregulated genes, while those in *Mycena* hyphae were notably decreased compared to pure culture, with fewer upregulated than downregulated genes. As histologically confirmed by Fan Li et al., once symbiosis is established, the seeds of *G. elata* begin to grow with increased biological activity, while the fungal hyphae are restricted and digested [43].

During the formation of protocorms, five genes (ZEP, evm.TU.scaffold_23.31; NCED-1, evm.TU.scaffold_79.140; NCED-2, evm.TU.scaffold_5.245; ABA2, evm.TU.scaffold_78.141; AAO3, evm.TU.scaffold_79.110) were found to be involved in ABA biosynthesis (Figure 4). However, the expression levels of these genes were relatively low and did not markedly change, except for 9-cis-epoxycarotenoid dioxygenase (NCED-2; evm.TU. scaffold_5.245), which was significantly downregulated to 6.08–16.65% compared to its expression in *Gastrodia elata* seeds (Table S6, Figure 4). In higher plants, the crucial role of ABA and related enzymes during the transition from seed maturation to germination has been extensively studied [44]. Carotenoid cleavage constitutes a key step in ABA biosynthesis, which is regulated by the NCED gene family [45]. In *Arabidopsis thaliana*, AtNCED6 is expressed specifically in the endosperm and AtNCED9 is expressed in both the embryo and endosperm; both genes are essential for inducing and maintaining seed dormancy [46]. In the orchid *Phaius tankervilliae*, PtNCED1 is directly involved in regulating ABA content in seed dormancy [47]. Lang et al. revealed that *Triticum aestivum* resisted pre-harvest sprouting when NCED was upregulated in ABA biosynthesis [48]. Our results suggest that significantly downregulated NCED-2 (evm.TU. scaffold_5.245) inhibits ABA biosynthesis, thereby reducing the inhibition of seed germination by ABA.

During the formation of protocorms, a total of 30 genes were found to be involved in ABA signal transduction (Figure 5). However, the expression levels of these genes were relatively low and did not notably change, except for PYL12-like (evm.TU.scaffold_61.29), which was significantly downregulated to 0.37–8.54% compared to its expression in *Gastrodia elata* seeds (Table S7, Figure 5). The PYL family, containing 14 members (PYR1 and PYL1–13), is the largest family of plant hormone receptors [49]. This family has been reported to be involved in ABA-mediated seed germination [32]. In *Arabidopsis*, PYR1-PYL1-PYL2-PYL4 [31], PYL12 [50], and PYL13 [51] act as positive regulators of ABA signaling during seed germination and early seedling development. Among these, PYL12 expression was found to be high in mature dry seeds but dropped sharply after germination [50]. This is consistent with our results, suggesting that significantly downregulated PYL12-like (evm.TU.scaffold_61.29) prevents ABA signal transduction and thereby breaks the dormancy of seeds. Miao et al. utilized CRISPR/Cas9 technology to edit PYL genes in rice. The single pyl mutant pyl12 exhibited significant defects in seed dormancy, causing rice seeds to germinate before harvest [52]. Inspired by their strategy, this study could provide a reference for future research aiming to improve the germination rate of *G. elata* seeds.

## 4. Materials and Methods

### 4.1. Symbiotic Germination of Gastrodia elata Seeds

The *Mycena* sp. strain WM (Guizhou Wumeng Fungi Industry Co., Ltd., Bijie, China) was incubated at 27 °C for two weeks with PDA medium (potato 200 g/L, glucose 20 g/L, agar 15 g/L). Mature capsules of *Gastrodia elata* were obtained from the Xiaocaoba Tianma production area in Zhaotong, Yunnan, China. The surfaces of mature capsules were sterilized with 70% ethyl alcohol for 1 min and 95% ethyl alcohol for 30 s and then briefly burned in flame. Seeds inside the capsules were immediately collected in a 1.5 mL microcentrifuge tube and maintained at 4 °C in a silica gel dryer [53]. Symbiotic germination of *G. elata* seeds with *Mycena* sp. WM hyphae was performed according to the description in a previous report [54]. Germination performance was observed daily under a stereomicroscope (DFC450, Leica), and images were captured digitally using a CCD camera attached to the stereomicroscope. The formation of protocorms served as the indicator of seed germination [55]. The protocorms were carefully collected in a 2 mL microcentrifuge tube under a stereomicroscope, washed with sterile water three times, dried, and then stored in a new 2 mL sterile microcentrifuge tube. For histological observation, *G. elata* seeds and protocorms were placed in formalin-aceto-alcohol (FAA) fixation solution. For transcriptomic studies, the sampling time points (5th, 10th, 15th, and 20th days) were determined according to histological observations. Samples of equal fresh weight of WM hyphae (M), *G. elata* seeds (d0), and protocorms (d5, d10, d15 and d20) were prepared in biological triplicates and immediately stored at −80 °C.

### 4.2. Histological Observation

The fixed samples were processed by the conventional paraffin embedding and sectioning method [56], with some modifications. A cold plate (HistoCore Arcadia C) was used for sample embedding, and a semiautomatic rotary microtome (Leica RM2245) was used to produce slices with a thickness of 3 μm. A glycogen PAS kit (G1281, Beijing SolarBio Science & Technology Co., Ltd., Beijing, China) was used for staining. The sections were observed and the images were captured digitally using a CCD camera attached to a light microscope (Axiao Cam MRc5, ZEISS).

### 4.3. Genome Sequencing, Assembly and Annotation

Total DNA of *Mycena* sp. WM was extracted with a modified cetyltrimethylammonium bromide protocol [57]. Sequencing, assembly and annotation were all performed at the Beijing Genomics Institute (BGI). The genome was sequenced using the BGI’s DNBseq™ technology platform. Clean data were assembled using SOAPdenovo2 short sequence assembly software [58,59]. The software Augustus 3.2.1 [60] was used for gene model prediction, and all gene annotations were completed by the software DIAMOND v2.0.7 [61] against seven databases (SWISS-PROT [62], COG [63], GO [64], KEGG [65], PHI [66], CAZy [67], and NCBI NR). The genomes of three strains of orchid mycorrhizal fungi—*Sebacina vermifera* (Accession No. JMDS00000000), *Tulasnella calospora* (Accession No. JMDT00000000) and *Ceratobasidium* sp. (Accession No. WITF00000000)—were retrieved from NCBI and annotated in the CAZy database [67]. The genomic sequences of *Gastrodia elata* were retrieved from the National Genomics Data Center (NGDC, Accession No. GWHAAEX00000000, https://bigd.big.ac.cn/gwh/Assembly/129, accessed on 21 September 2020).

### 4.4. Transcriptome Sequencing, Mapping, Assembly, and Annotation

Total RNA was extracted from the samples using TRIzol^®^ Reagent (Invitrogen, Carlsbad, CA, USA). Transcriptome sequencing was performed using the Illumina HiSeq platform. After quality control and cleaning of raw data, the clean data were mapped to the reference genomes JAHHPV000000000 (*Mycena* sp. WM) and GWHAAEX00000000 (*Gastrodia elata*) with the software HISAT2 [68,69]. The mapped data were assembled using the software Cufflinks 2.2.1 [70]. Genes/transcripts were annotated in NCBI NR, SWISS-PROT [62], COG [63], GO [64], KEGG [65], and Pfam (http://pfam.xfam.org/, accessed on 28 October 2020) databases.

### 4.5. Differential Gene Expression Analysis

The genomic annotation file was fed into the software RSEM v1.3.1 [71] to obtain read counts of each sample gene, which were further converted to standardized gene expression levels with the fragments per kilobase per million mapped reads (FPKM) algorithm. DEseq2 [72] was used to analyze the differentially expressed genes, with screening criteria of fold change (FC) of ≥2 and false discovery rate (FDR) of <0.01. The DEGs were then analyzed using a Venn diagram, GO [64] functional classification, and KEGG [65] functional enrichment to obtain their main functions and metabolic pathways.

### 4.6. Quantifying Expression Levels of Target Genes by RT-qPCR

The RNA applied for transcriptome sequencing was used for quantitative PCR analyses. Primers designed with Primer Premier 5.0 [73] are shown in Table S8. The primers were synthesized by Tsingke Biotechnology Co., Ltd., Beijing, China. The Fastking gDNA Dispelling RT SuperMix (KR118-01, Tiangen Biotech Co., Ltd., Beijing, China) was used for reverse transcription. RT-qPCR with three biological replicates and three experimental replicates and calculations of relative mRNA levels were performed as previously described [74]. The 18S rRNA [74] from *Gastrodia elata* and glyceraldehyde-3-phosphate dehydrogenase (GADPH) [75] from *Mycena* sp. WM were used as reference loci for the normalization of gene expression.

## 5. Conclusions

The genome assembly of *Mycena* sp. WM was conducted in this study, and the genome of *Gastrodia elata* was retrieved from NGDC. Transcriptomes obtained during their coculture were mapped to their genomes separately. Four (MnP-short: GME42162_g, GME24208_g; laccase: GME40562_g, GME8664_g) of the 25 lignin-modifying enzymes in *Mycena* were significantly upregulated. It is suggested that *Mycena* secretes MnP and laccases that decompose the lignin seed coats of *G. elata*. As many as 2860 genes related to reduced virulence and loss of pathogenicity were annotated in *Mycena*. It is hypothesized that these genes contribute to the symbiosis between *G. elata* and *Mycena*. When cocultured, more genes were upregulated in *G. elata* while more were downregulated in *Mycena*. These results imply that the biological activity of *G. elata* seeds was slightly increased after the initiation of germination, while that of *Mycena* hyphae was notably decreased. When the symbiotic relationship was established between *G. elata* and *Mycena*, NCED-2 and PYL12-like genes, which are involved in ABA biosynthesis and signal transduction, respectively, were downregulated to break seed dormancy. This study identified key factors in lignin degradation by *Mycena* and in ABA biosynthesis and signaling in *G. elata* seeds, providing a reference for studies on the molecular mechanism of symbiosis between orchids and fungi.

## Figures and Tables

**Table 1:** Transcriptome expression levels (FPKM) of 25 lignin-modifying enzyme genes on the 5th day (d5) of the coculture versus the pure culture (M) of *Mycena* sp. WM.

Gene ID	Description	d5/M	*p* Value	Regulation
GME18701_g	Manganese peroxidase	1.72	0.542	Up
GME20_g	Manganese peroxidase isozyme precursor	0	0.018	Down
GME35282_g	Manganese peroxidase 3	1.8	0.053	Up
GME35281_g	Manganese peroxidase 3	0.22	0	Down
GME42220_g	Manganese peroxidase 1 precursor	-	-	-
GME26993_g	Manganese peroxidase 1 precursor	0.06	0.011	Down
GME4917_g	MnP-short, short manganese peroxidase	0.47	0.615	Down
GME24904_g	Manganese peroxidase 3	2.14	0.106	Up
GME42162_g	MnP-short, short manganese peroxidase	7.01	0.033	Up
GME24208_g	MnP-short, short manganese peroxidase	5.92	0.040	Up
GME43588_g	Manganese peroxidase 3	-	-	-
GME35915_g	Versatile peroxidase	0.81	0.201	Down
GME16021_g	Laccase 2 precursor	0.02	0.069	Down
GME1615_g	Laccase	1.74	0.435	Up
GME27679_g	Laccase	0.16	0.107	Down
GME40562_g	Laccase-1	12.58	0.022	Up
GME48196_g	Laccase	-	-	-
GME5711_g	Laccase-1	0.12	0.227	Down
GME6073_g	Putative laccase 2t, partial	0.01	0.138	Down
GME6075_g	Laccase-1	0	0.423	Down
GME6393_g	Laccase	0.05	0.01	Down
GME7185_g	Laccase, partial	0.19	0.17	Down
GME8664_g	Laccase	7.53	0.007	Up
GME23003_g	Dye-decolorizing peroxidase;	2.81	0.072	Up
GME6106_g	DyP-type peroxidase	-	-	-

## Data Availability

The genome and transcriptome data have been deposited at the National Center for Biotechnology Information Sequence Read Archive under accession numbers PRJNA730200 and PRJNA729600, respectively.

## Supplementary Materials

The following are available online at https://www.mdpi.com/article/10.3390/ijms22126557/s1.
