# Supplementary material for: Transcriptome Reveals Roles of Lignin-Modifying Enzymes and Abscisic Acid in the Symbiosis of Mycena and Gastrodia elata
Source: Int J Mol Sci. 2021 Jun 18;22(12):6557. doi: 10.3390/ijms22126557 (PMC8235111; doi:10.3390/ijms22126557)
Supplement: Supplementary file 1 [file ijms-22-06557-s001.zip › ijms-1262219-supplementary.pdf]

International Journal of
Molecular Sciences

MDPI

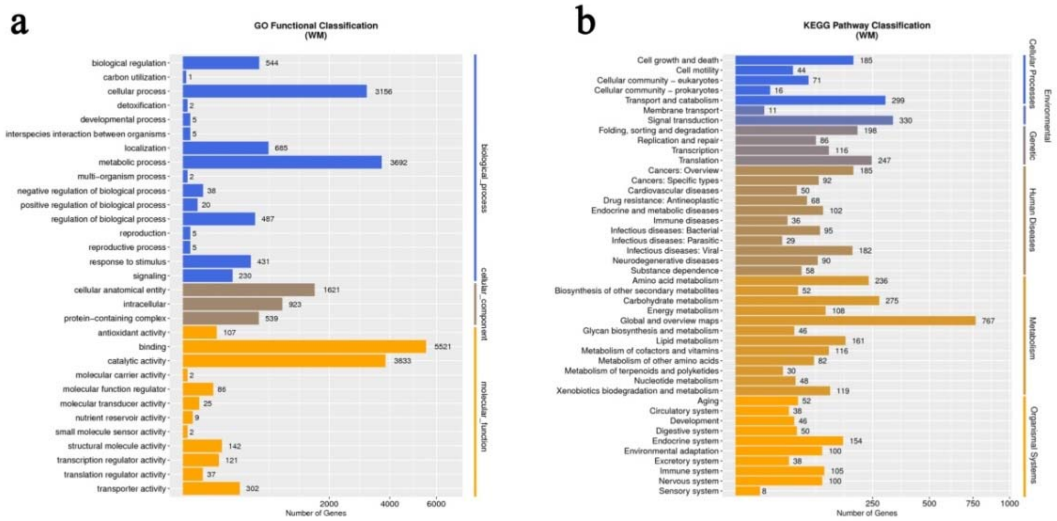

Figure S1. GO (**a**) and KEGG (**b**) classification of predicted genes in *Mycena* sp. WM.

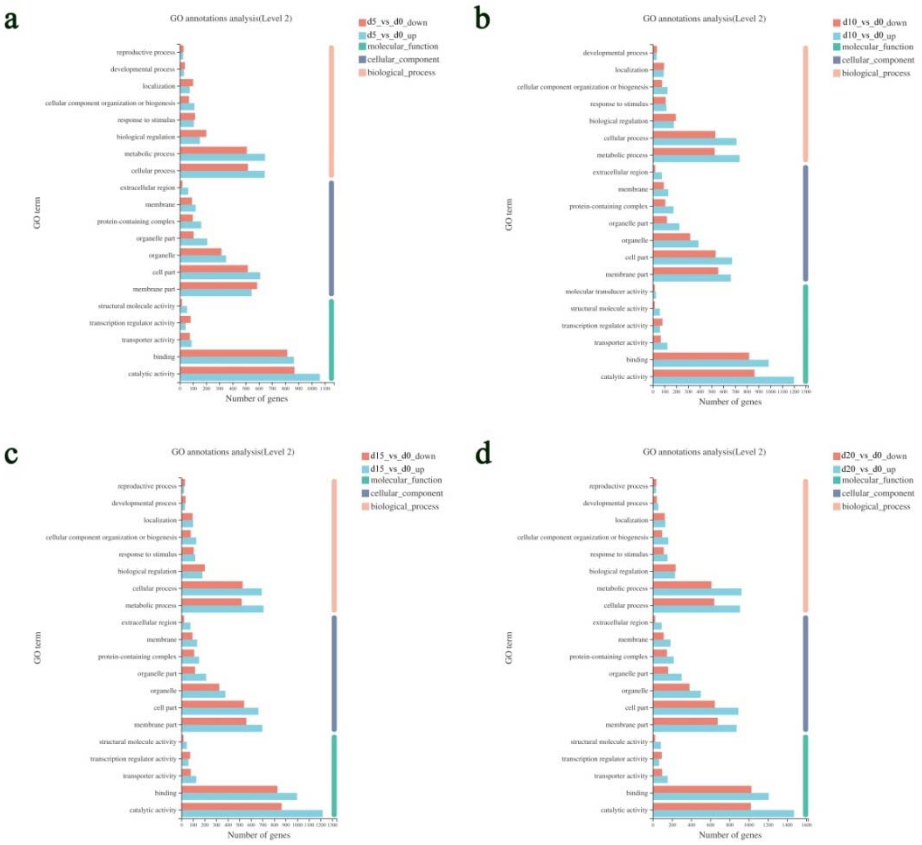

Figure S2. GO annotation of up-/down-regulated DEGs in *Gastrodia elata* seeds/protocorms. (**a**) DEGs on the 5th (d5) day of the co-culture compared with the initial expression (d0); (**b**) DEGs on the 10th (d10) day of the co-culture compared with the initial expression (d0); (**c**) DEGs on the 15th (d15) day of the co-culture compared with the initial expression (d0); (**d**) DEGs on the 20th (d20) day of the co-culture compared with the initial expression (d0).

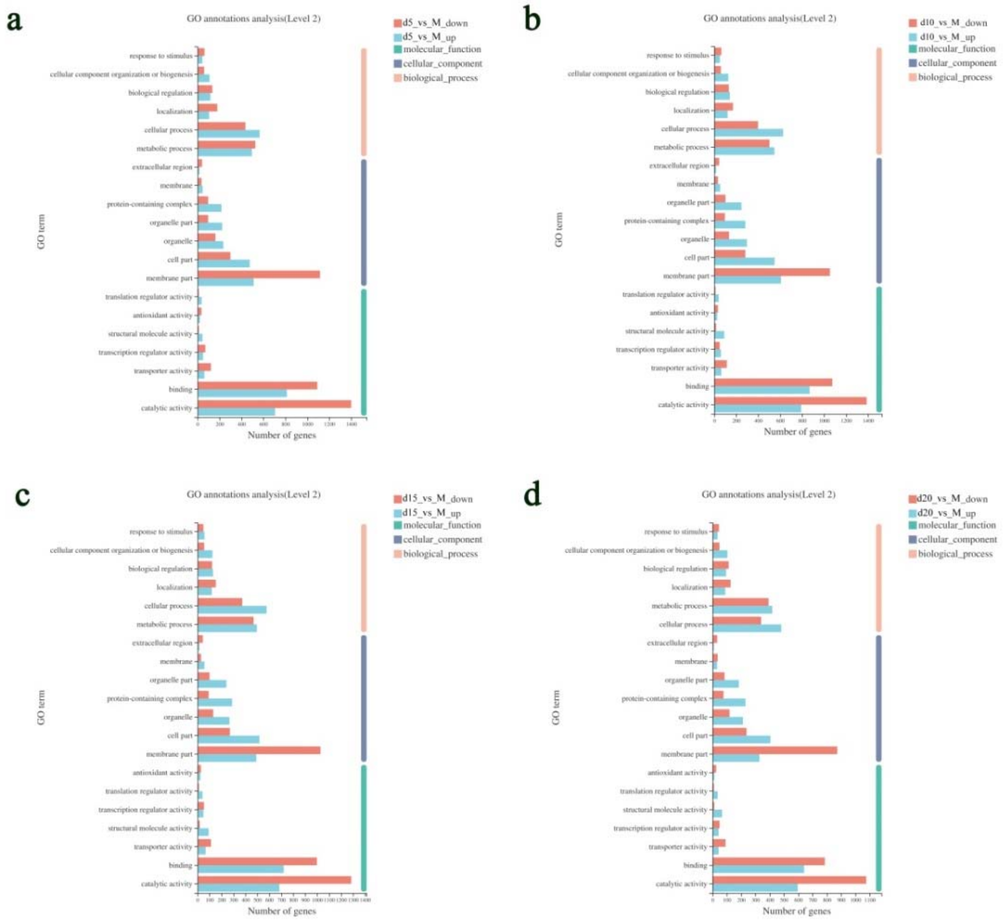

Figure S3. GO annotation of up-/down-regulated DEGs in *Mycena* sp. WM. (**a**) DEGs on the 5th (d5) day of the co-culture compared with the initial expression (M); (**b**) DEGs on the 10th (d10) day of the co-culture compared with the initial expression (M); (**c**) DEGs on the 15th (d15) day of the co-culture compared with the initial expression (M); (**d**) DEGs on the 20th (d20) day of the co-culture compared with the initial expression (M).

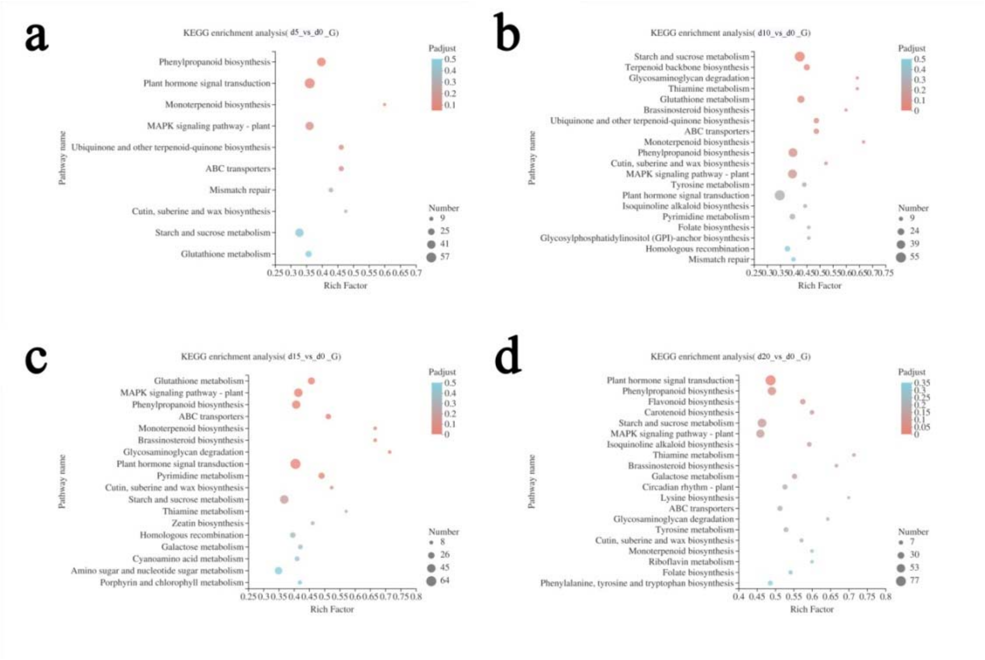

Figure S4. KEGG enrichment of DEGs in *Gastrodia elata* seeds/protocorms. (**a**) DEGs on the 5th (d5) day of the co-culture compared with the initial expression (d0); (**b**) DEGs on the 10th (d10) day of the co-culture compared with the initial expression (d0); (**c**) DEGs on the 15th (d15) day of the co-culture compared with the initial expression (d0); (**d**) DEGs on the 20th (d20) day of the co-culture compared with the initial expression (d0).

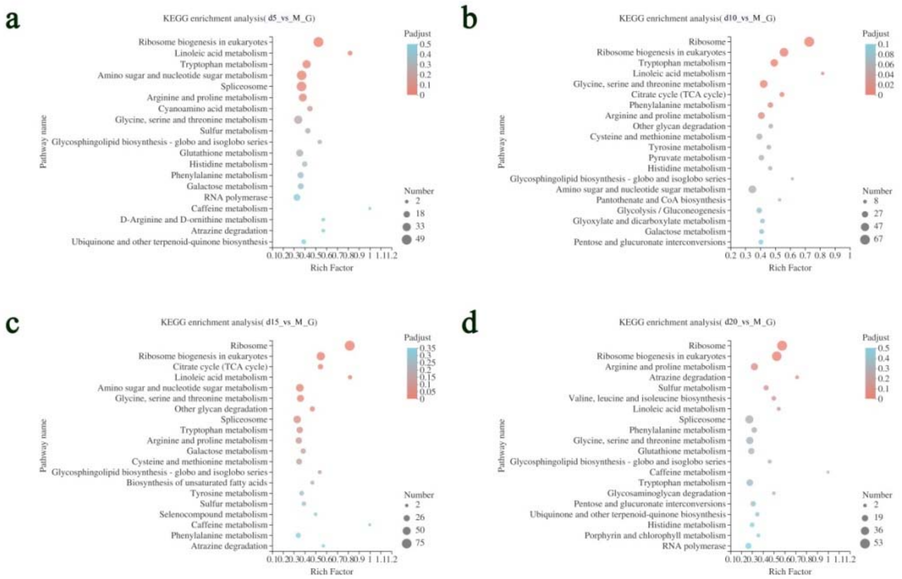

Figure S5: KEGG enrichment of DEGs in *Mycena* sp. WM. (**a**) DEGs on the 5th (d5) day of the co-culture compared with the initial expression (M); (**b**) DEGs on the 10th (d10) day of the co-culture compared with the initial expression (M); (**c**) DEGs on the 15th (d15) day of the co-culture compared with the initial expression (M); (**d**) DEGs on the 20th (d20) day of the co-culture compared with the initial expression (M).

Table S1. Gene numbers of different categories in *Mycena* sp. WM annotated against the CAZy database.

CAZy	Number
Glycoside hydrolases (GHs)	202
Glycosyl transferases (GTs)	53
Polysaccharide lyases (PLs)	16
Carbohydrate esterases (CEs)	35
Auxiliary activities (AAs)	118
Cellulose-binding module (CBM)	21

Table S2. Phenotypes and numbers of genes linked to the pathogen–host interaction in *Mycena* sp. WM.

Phenotypes	Numbers	Proportions
Unaffected pathogenicity	1,733	34.49%
Reduced virulence	2,396	47.69%
Loss of pathogenicity	464	9.24%
Increased pathogenicity	181	3.60%
Effector	26	0.52%
Lethal	213	4.24%
Target resistance to chemical	11	0.22%
Total	5,024	100%

Table S3. Pure culture and co-culture transcriptome information mapping to *Gastrodia elata* and *Mycena* sp. WM.

		Gastrodia elata	*Mycena* sp. WM
**Sample***	**Clean reads**	**Mapped reads (percentages)**	**Mapped reads (percentages)**
M_1	43,638,916	-	35,746,506（81.91%）
M_2	42,229,818		34,061,778（80.66%）
M_3	42,479,662	-	34,996,974（82.39%）
d0_1	44,547,638	43,004,820（96.54%）	-
d0_2	43,513,882	41,811,079（96.09%）	-
d0_3	45,153,866	43,318,285（95.93%）	-
d5_1	73,707,120	32,653,260（44.30%）	31,496,836（42.73%）
d5_2	72,516,568	31,934,907（44.04%）	31,392,312（43.29%）
d5_3	74,035,894	40,614,381（54.86%）	25,356,820（34.25%）
d10_1	74,438,914	17,657,663（23.72%）	45,562,134（61.21%）
d10_2	69,627,478	17,933,333（25.76%）	41,073,780（58.99%）
d10_3	72,358,844	13,694,331（18.93%）	46,738,408（64.59%）
d15_1	75,452,480	31,798,364（42.14%）	33,551,274（44.47%）
d15_2	70,269,938	19,920,576（28.35%）	39,393,584（56.06%）
d15_3	74,895,108	16,957,873（22.64%）	46,067,372（61.51%）
d20_1	74,383,298	62,879,371（84.53%）	7,002,274（9.41%）
d20_2	72,989,760	59,996,787（82.20%）	82,032,34（11.24%）
d20_3	72,386,600	54,368,273（75.11%）	12,4188,04（17.16%）

* Sample M represents the pure *Mycena* sp. WM; Sample d0 represents the pure *Gastrodia elata* seed; Samples d5, d10, d15 and d20 represent their co-culture collected on the 5th, 10th, 15th and 20th days, respectively. Three replicates were performed for all samples (_1, _2 and _3).

Table S4. The number of expressed genes that were annotated against each database.

Database	*Gastrodia elata*	*Mycena* sp. WM
	**Numbers (percentages)**	**Numbers (percentages)**
GO	12,267 (69.5%)	15,265 (54.18%)
KEGG	69,86 (39.58%)	5,662 (20.1%)
COG	14,264 (80.82%)	3,441 (12.21%)
NR	15,124 (85.69%)	20,000 (70.99%)
SWISS-PROT	12,334 (69.88%)	8,028 (28.5%)
Pfam	12,500 (70.82%)	11,273 (40.01%)
Total	15,186 (86.04%)	20,486 (72.72%)

Table S5. Numbers of lignin-degrading enzyme genes in mycorrhizal fungi of orchids.

Specific name	POD	Laccases	DyP
Ceratobasidium sp.	0	10	5
Serendipita vermifera	0	5	2
Tulasnella calospora	0	1	2
Mycena sp. WM	12	11	2

Table S6. Expression levels of genes related to abscisic acid (ABA) biosynthesis in *Gastrodia elata*.

Gene ID	Gene name	Description	d0	d5/d0	d10/d0	d15/d0	d20/d0
evm.TU.scaffold_23.31	ZEP	zeaxanthin epoxidase, chloroplastic	0.26	2774.68%	3692.41%	3917.72%	5313.92%
evm.TU.scaffold_79.140	NCED-1	9-cis-epoxycarotenoid dioxygenase,	2.51	62.73%	64.85%	149.73%	84.35%
evm.TU.scaffold_5.245	NCED-2	9-cis-epoxycarotenoid dioxygenase	24.25	13.65%	16.65%	7.45%	6.08%
evm.TU.scaffold_78.141	ABA2	zerumbone synthase	9.22	236.84%	182.10%	151.01%	90.20%
evm.TU.scaffold_79.110	AAO3	indole-3-acetaldehyde oxidase	0.87	139.85%	160.54%	192.34%	240.61%

Table S7. Expression levels of genes related to abscisic acid (ABA) signaling in *Gastrodia elata*.

Gene ID	Gene name	Description	d0	d5/d0	d10/d0	d15/d0	d20/d0
evm.TU.scaffold_108.9	PYR1	ABA receptor PYR1	1.09	159.94%	143.43%	157.19%	280.12%
evm.TU.scaffold_80.139	PYL4 -like	ABA receptor PYL4-like	27.95	122.20%	80.83%	72.20%	43.58%
evm.TU.scaffold_60.4	PYR1-1	ABA receptor PYR1	1.18	152.69%	237.68%	269.97%	633.14%
evm.TU.scaffold_89.78	PYL8 -like	ABA receptor PYL8-like	22.93	182.55%	188.37%	166.01%	165.92%
evm.TU.scaffold_61.29	PYL12 -like	ABA receptor PYL12-like	1547.07	7.15%	6.66%	8.54%	0.37%
evm.TU.scaffold_129.56	PP2C 68	protein phosphatase 2C 68	38.32	8.58%	19.99%	12.95%	2.27%
evm.TU.scaffold_1.9	PP2C 51	protein phosphatase 2C 51	198.65	25.06%	33.17%	34.79%	23.41%
evm.TU.scaffold_78.217	PP2C 37-like	protein phosphatase 2C 37-like	11.16	169.20%	230.09%	158.71%	225.75%
evm.TU.scaffold_100.47	PP2C 50-like	protein phosphatase 2C 50-like	4.45	581.81%	801.42%	729.27%	567.74%
evm.TU.scaffold_0.483	PP2C 6	protein phosphatase 2C 6	37.90	158.40%	173.84%	153.96%	116.85%
evm.TU.scaffold_43.38	SnRK2_ SAPK7 -like	serine/threonine-protein kinase SAPK7-like	0.00	-	-	-	-
evm.TU.scaffold_44.235	SnRK2_SAPK7-like-1	serine/threonine-protein kinase SAPK7-like	109.62	126.02%	130.60%	113.94%	98.76%
evm.TU.scaffold_148.69	SnRK2_ SAPK3	serine/threonine-protein kinase SAPK3 isoform X1	119.63	27.67%	36.37%	33.25%	12.47%
evm.TU.scaffold_139.37	SnRK2_ SAPK7 -like-2	serine/threonine-protein kinase SAPK7-like	2.57	845.01%	1107.13%	1040.21%	1785.21%
evm.TU.scaffold_127.76	SnRK2_SAPK2 -like	serine/threonine-protein kinase SAPK2-like	2.62	748.28%	1116.77%	1133.93%	952.60%
evm.TU.scaffold_51.33	SnRK2_SAPK10	serine/threonine-protein kinase SAPK10	11.22	275.20%	301.84%	322.33%	319.57%
evm.TU.scaffold_34.62	ABF_TRAB1	bZIP transcription factor TRAB1	0.36	0.00%	0.00%	0.00%	76.15%
evm.TU.scaffold_63.37	ABF_ABI5-like	ABA -insensitive 5-like protein 5	1.37	21.36%	115.05%	62.86%	47.57%
evm.TU.scaffold_50.162	ABF_TRAB1-1	bZIP transcription factor TRAB1	14.16	28.63%	52.54%	60.73%	47.50%
evm.TU.scaffold_93.33	ABF_TRAB1-like-1	bZIP transcription factor TRAB1-like	7.19	183.97%	248.19%	234.29%	313.35%
evm.TU.scaffold_13.172	ABF-1	bZIP transcription factor 12	16.31	192.97%	177.76%	188.47%	155.49%
evm.TU.scaffold_263.11	ABF_ABI5-like-1	ABA -insensitive 5-like protein	3.12	6.63%	36.26%	13.05%	0.00%
evm.TU.scaffold_45.126	ABF-2	bZIP transcription factor 12-like	1.66	358.23%	1087.95%	961.24%	1710.24%
evm.TU.scaffold_99.130	ABF_ABI5-like-2	ABA -insensitive 5-like protein 5	0.22	0.00%	0.00%	0.00%	740.30%
evm.TU.scaffold_34.59	ABF_ABI5-like-3	ABA -insensitive 5-like protein 5	0.00	-	-	-	-
evm.TU.scaffold_0.366	ABF_ABI5-like-4	ABA -insensitive 5-like protein 2	2.46	792.56%	1539.51%	1701.62%	1371.58%
evm.TU.scaffold_57.111	ABF_TRAB1-3	bZIP transcription factor TRAB1	0.10	180.00%	56.67%	0.00%	146.67%
evm.TU.scaffold_91.3	ABF_ABI5-like-5	ABA -insensitive 5-like protein 2	13.64	137.78%	137.76%	184.41%	136.41%

Table S8. The quantitative reverse transcription PCR primers of putative genes.

**Gene ID**	**Forward primers (5′-3′)**	**Reverse primers (5′-3′)**
18S rDNA	GCTTTGGAGCGTGAGGTAGA	CTTGCCCGCATAGCACAAC
evm.TU.scaffold_61.29	ATCTCAGCCGTCCGATTCCC	CTGAGCGAGCACGATTTCAC
evm.TU.scaffold_5.245	TTTCTGGAATGCGTGGGAGG	AATCTCAACCAGCTCGCTCC
GADPH	ATGCCTTTTCCAACACCCGA	TAGTATGTCGGCGCGTAAGGT
GME42162_g	GTCCCCGAGCCCTTTGATAC	GGCTGCGATTGTGTGGGA
GME24208_g	TTGTCGCTCTGCTTTCGTCT	GAGCTGAACCTCGATGAAAACC
GME40562_g	AACGAGCGACAACACTACCA	GGTAGGATGCACGGAGTTGG
GME8664_g	ACATTCCCGACGAACGAACA	TCGACATCATAGAGAGACGCA
